# Spring onion seed demand forecasting using a hybrid Holt-Winters and support vector machine model

**DOI:** 10.1371/journal.pone.0219889

**Published:** 2019-07-25

**Authors:** Yihang Zhu, Yinglei Zhao, Jingjin Zhang, Na Geng, Danfeng Huang

**Affiliations:** 1 Dept. of Plant Science, School of Agriculture & Biology, Shanghai Jiao Tong University, Shanghai, People’s Republic of China; 2 Dept. of Industrial Engineering & Management, Shanghai Jiao Tong University, Shanghai, People’s Republic of China; Jawaharlal Nehru University, INDIA

## Abstract

Demand for spring onion seeds is variable and maintaining its supply is crucial to the success of seed companies. Spring onion seed demand forecasting, which can help reduce the high operational costs increased by long-period propagation and complex logistics, has not previously been investigated yet. This paper provides a novel perspective on spring onion seed demand forecasting and proposes a hybrid Holt-Winters and support vector machine (SVM) forecasting model. The model uses dynamic factors, including historical seed sales, seed inventory, spring onion crop market price and weather data, as inputs to forecast spring onion seed demand. Forecasting error, i.e. the difference between actual and forecasted demand, is assessed. Two advanced machine learning models are trained on the same dataset as benchmark models. Numerical experiments using actual commercial sales data for three spring onion seed varieties show the proposed hybrid model outperformed the statistical-based models for all three forecasting errors. Seed inventory, spring onion crop market price and historical seed sales are the most important dynamic factors, among which seed inventory has short-term influence while other two have mid-term influence on seed demand forecasting. The absolute minimum temperature is the only factor having long-term influence. This study provides a promising spring onion seed demand forecasting model that helps understand the relationships between seed demand and other dynamic factors and the model could potentially be applied to demand forecasting of other crop seeds to reduce total operational costs.

## Introduction

Spring onion (*Allium fistulosum L*., also known as Welsh onion or scallion) probably originated in north-western China and is widely cultivated throughout South-East Asia and Europe. Spring onion maintains vegetative growth all year round, except in winter, and is commercially grown as an annual that is usually sown and/or transplanted as seedlings in early spring, summer or autumn. The majority of spring onion seeds are bought by seedling companies, agents or growers’ cooperatives.

**Fig 1:**
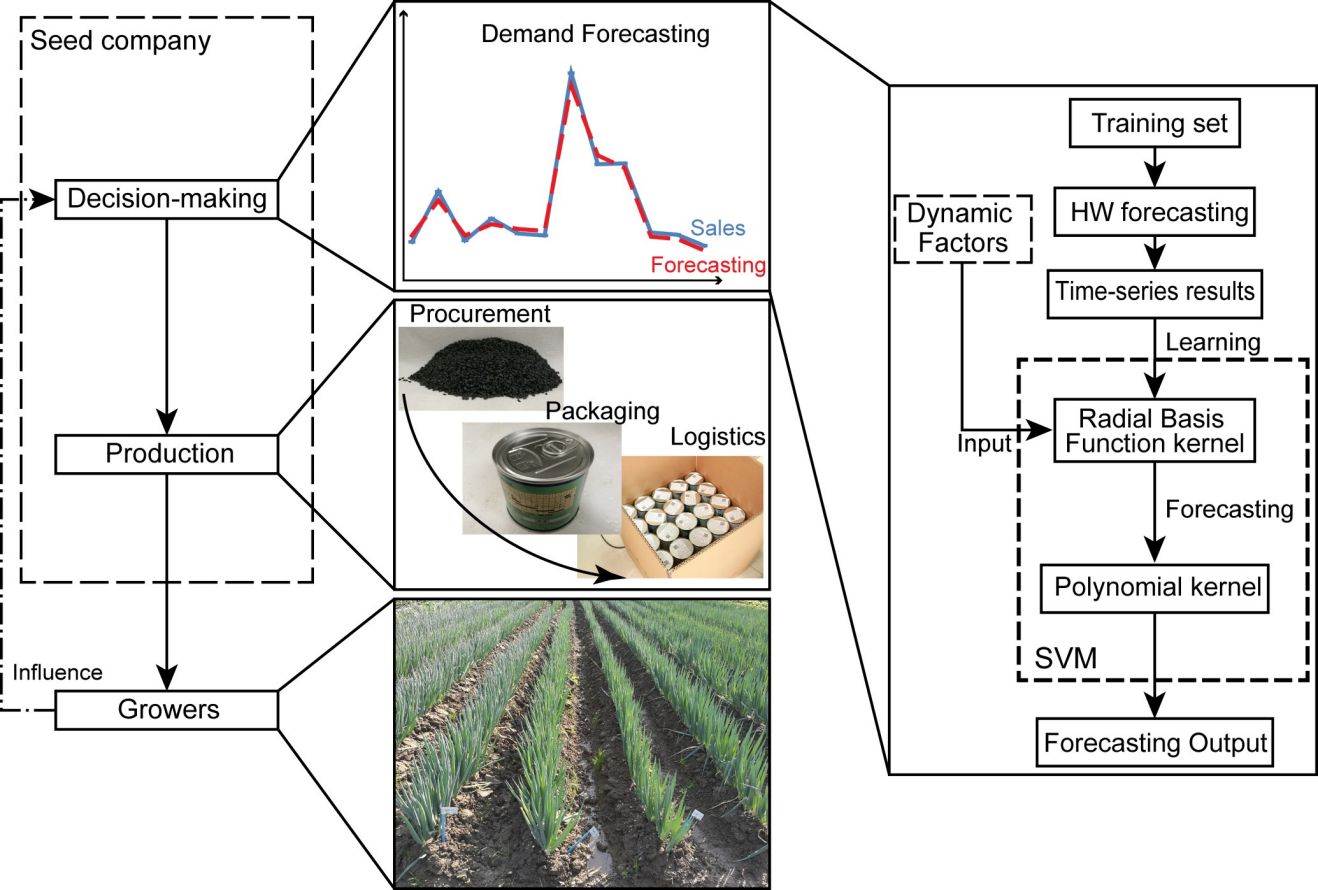
Spring onion seed demand forecasting methodology and its importance. The left side shows the relationships between demand forecasting and other processes. The right side shows the hybrid Holt-Winters (HW) and support vector machine (SVM) demand forecasting methodology proposed in this study.

There is large demand for spring onion seeds in China. Seed companies aim to provide a reliable supply of seed in the right quantities at the right time to growers. However, seed procurement, storage, packaging and logistics are complicated because of various reasons such as marketing, supply chain and seed deterioration. They require seed companies use significant manpower and finance to maintain their inventory to satisfy their customers, which leads to high operational costs. Moreover, it usually takes three years to propagate spring onion seeds from the parent plant, highlighting the importance of demand forecasting during seed production planning. Therefore, accurate demand forecasting is crucial for inventory control, in order to reduce operational costs and ensure market share growth for seed companies, as well as ensuring sufficient seed supply for customers and growers. It is essential for seed companies to develop more accurate demand forecasting methods for spring onion seeds (Fig 1).

Since 1990, both empirical and statistical-based models have been developed in an attempt to forecast demand for a variety of crop seeds and other agricultural products. However, the performance of these models varies dramatically from case to case, as various factors affect demand, such as weather conditions, crop prices, seed inventory, and even growers’ preferences [1].

Although the term ‘demand’ is used when referring to forecasting in this paper, the actual demand is usually considered unknown and sales information is used as an approximation of demand [2]. Thus, sales information is used to forecast future demand, and the terms ‘demand’ and ‘sales’ are used interchangeably.

Since demand forecasting problems were first proposed and studied in 1970’s, numerous statistical-based models such as autoregressive integrated moving average (ARIMA), seasonal ARIMA [3], Holt-Winters [4] and other regression models have been developed. Many researchers have attempted to forecast demand in agriculture using time-series and statistical-based models, such as models for forecasting wheat production [5] or rice yield [6] based on historical yield and weather data. The forecasting errors were 32.5%-41.6% and 23.6%-25.7%, respectively. Naidu (2015) used historical sales data and an ARIMA model to forecast wholesale data for potato and onion crops, for which the forecasting errors were 28.30% and 29.51%, respectively [7]. Da Veiga et al. (2014) found the Holt-Winters model performed well for forecasting the food demand [8]. The forecasting errors were 14.97%-15.66% among various products. Researches showed that the forecasting performance of statistical-based models is unstable when applied to actual data, as statistical-based models employ hard computing based on exact models, and most are based on linear analysis [9]. However, the demand for agricultural products is usually affected by many non-linear factors, which can significantly reduce the accuracy of statistical-based models.

The use of advanced machine learning models in forecasting has developed rapidly. For example, an artificial neural network (ANN) was used to forecast water resource variables in river systems [10]. The forecasting errors were smaller than 18.00% and the authors suggested the input independence should be high in order to reduce model outputs uncertainty. Fuzzy rule-based systems were used to predict storage times for pork based on five pork quality parameters with the forecasting accuracy of 93.93%-94.41% [11]. A random forest (RF) model was used to predict sugarcane yield based on simulated biomass indices, observed climate and seasonal climate prediction indices [12]. The forecasting accuracy reached 95.45%. A support vector machine (SVM) approach was used to forecast sales of five computer products based on weekly sales data and the forecasting errors were 4.09%-8.62% [13]. Compared with statistical-based models, whose forecasting error is difficult to be lower than 20%, all these advanced machine learning models have demonstrated better performance in terms of forecasting error or accuracy within their specific contexts.

The non-linearity of ANN models, which are simulated from biological systems, enables more accurate demand forecasting compared to statistical-based models [14]. Several researchers have used advanced machine learning models to address agricultural forecasting problems. Co and Boosarawongse (2007) found an ANN performed well in forecasting the weekly export price of Thai rice, but could not explain how agricultural and environmental factors influence the price of rice [15]. In general, the accuracy and robustness of statistical-based models in agricultural contexts vary dramatically from case to case, and advanced machine learning models generally outperform statistical-based models in most cases. However, Fortin et al. (2011) suggested advanced machine learning models may not replace previous statistical-based models, and a good forecasting model should not completely abandon statistical methods [16].

The SVM approach was first proposed as a new statistical learning tool for pattern classification by Boser et al. in 1992 [17]. SVM models have achieved higher accuracy and provided global optimal solutions with fewer over-fitting problems than ANN models in many areas of research [18]. Unlike ANN models, which follow the empirical risk minimization principle, SVM models try to minimize the upper bound of the generalization error rather than minimizing the training error; this approach is called the structural risk minimization principle. SVM models offer the advantage of converting complex nonlinear regression problems into linear regression problems in high dimensional feature space, which is useful for forecasting problems [13].

Since the introduction of the ε-insensitive loss function, SVM models have been extended to solve regression problems and become another important tool for forecasting problems [19]. Many studies of demand forecasting in areas outside agriculture have demonstrated the outstanding performance of SVM models based on different kernel functions compared to ANN models and other models [20]. Kumar and Thenmozhi (2014) also found SVM models outperformed other models and stated SVM have excellent capability to be used to create hybrid forecasting models with statistical-based models [21]. In agricultural research, SVM has been widely applied in image and sensor data detection [22]. However, SVM applications have not yet been reported for crop product or seed demand forecasting.

Spring onion seed demand forecasting has huge impact on the seed processing and marketing. Thus, the main objective of this paper is to compare the performances of statistical-based methods (ARIMA and Holt-Winters), advanced machine learning methods (ANN, random forest and SVM) and the hybrid model in forecasting the demand for spring onion seeds based on dynamic factors including historical sales, seed inventory, spring onion price and weather data. Secondly, the optimal and most accurate method for spring onion seed demand forecasting is proposed and the influences of dynamic factors on forecasting are discussed. Finally, the contributions, perspectives and remarks are presented.

## Materials and methods

### ARIMA model

The ARIMA model, also known as the Box-Jenkins model [3], has been a popular forecasting model since the late 1970’s. The key hypothesis in the ARIMA model is that the future value of the time series is a linear combination of past values and errors. The model is expressed as follows:

$$\begin{array}{cc} D_{t}= & a_{0}+a_{1}D_{t-1}+a_{2}D_{t-2}+\cdots +a_{p}D_{t-p}\\ & +\varepsilon_{t}-b_{1}\varepsilon_{t-1}-b_{2}\varepsilon_{t-2}-\cdots -b_{q}\varepsilon_{t-q} \end{array}$$ (1)

where, *D*_*t*_ is the actual value of demand and *ε*_*t*_ is the random error at time *t*, *a*_*i*_ and *b*_*i*_ are coefficients, and *p* and *q* are integers called the autoregressive and moving average parameters, respectively. The ARIMA model is a linear data-based approach that adapts parameters from the actual time series data. Therefore, non-linearity in the data significantly affects the performance of the ARIMA model; hybrid ARIMA and advanced machine learning models are widely proposed to be able to deal with non-linear data [23].

### Holt-Winters model

The Holt-Winters model is a speculation smoothing-based forecasting technique proposed in 1960 by Holt and Winters [24]. Unlike ARIMA, which uses parameters in the equations to address the seasonal trend in the original data, Holt's linear equations have a built-in seasonal factor equation that can capture the seasonality directly. The Holt-Winters model is widely applied to time series that show seasonal increases and decreases. Three smoothing equations are designed to calculate and estimate the deseasonalized series, trend and seasonal factors. Unlike ARIMA, Holt-Winters forecast values are generated from iterative steps, instead of calculations based on fitting to statistical models. There are two methods of seasonal factor modeling: additive and multiplicative Holt-Winters models. Although the multiplicative Holt-Winters (mul-HW) model cannot be utilized on data with null or negative values, it is incompatible with ARIMA and necessary to use. Nevertheless, the additive trend and seasonality found by the additive Holt-Winters model are covered by the output of ARIMA models [25] and it is not considered. The multiplicative Holt-Winters equations are:

$$Series:S_{t}=\alpha \frac{D_{t}}{c_{t-N}}+\left(1-\alpha \right)\left(S_{t-1}+G_{t-1}\right)$$ (2)

$$trend:G_{t}=\beta \left(S_{t}-S_{t-1}\right)+\left(1-\beta \right)G_{t-1}$$ (3)

$$Seasonal\;factors:c_{t}=\gamma \frac{D_{t}}{S_{t}}+\left(1-\gamma \right)c_{t-N}$$ (4)

$$Forecast:f_{t+m}=\left(S_{t}+G_{t}m\right)c_{t-N+m}$$ (5)

where, *N* is the length of the seasonal cycle, *D*_*t*_ is the actual value of demand, *S*_*t*_ is the deseasonalized series, *G*_*t*_ is the trend, *c*_*t*_ is the seasonal factor, *f*_*t+m*_ is the forecast value for *m* periods ahead, and *α*, *β* and *γ* are smoothing constants that are theoretically between 0 and 1. Since there are no general rules for choosing the smoothing constants and large smoothing constants will result in less stable forecasts, the optimal values are obtained via iteration by minimizing the squared one-step prediction error [26].

### SVM model

As previously stated, SVM models are based on the structural risk minimization principle, and attempt to minimize the upper bound of the generalization error rather than minimizing the training error [13]. SVM maps data in a non-linear manner onto a high-dimensional feature space and conducts linear regression in this space. The regression function is:

y=ωφ(X)+b (6)

where, *φ(****X****)* is the feature for which data are non-linearly mapped into space ***X***. The coefficients ***ω*** and *b* are estimated by minimizing the risk function, *R(C)*:

$$Minimize\;R\left(C\right)=C\frac{1}{N}\sum_{i=1}^{N}L_{\varepsilon }\left(d_{i},y_{i}\right)+\frac{1}{2}{\left\|\omega \right\|}^{2}$$ (7)

$$s.t.\;L_{\varepsilon }\left(d,y\right)=\{\begin{array}{l} \left|d-y\right|-\varepsilon ,\;\left|d-y\right|\geq \varepsilon \\ 0,\;\left|d-y\right|<\varepsilon \end{array}$$ (8)

where, *d*_*i*_ is the actual demand value in period *i*, *N* is the length of total data. *C* is the regularized constant determining the trade-off between the empirical error and the regularization term, and *ε* is a prescribed parameter that determines the upper bound of the error penalty. *L*_*ε*_(*d*,*y*) is called the ε-insensitive loss function. The first term in Eq (7) is the empirical error and the second term is used to measure the function flatness.

By introducing Lagrange multipliers, *α*_*i*_, *α*_i_^*^ (*α*_*i*_*α*_i_^*^ = 0, *α*_*i*_, *α*_i_^*^ ≥ 0), and letting the partial derivatives of ***ω***, *b* and *ζ*_i_^*^ equal zero, the problem can be expressed as:

$$\begin{array}{ll} Maximize\;R\left(\alpha_{i}-\alpha_{i}^{*}\right)= & \sum_{i=1}^{N}d_{i}\left(\alpha_{i}-\alpha_{i}^{*}\right)-\varepsilon \sum_{i=1}^{N}\left(\alpha_{i}+\alpha_{i}^{*}\right)\\ & -\frac{1}{2}\sum_{i=1}^{N}\sum_{j=1}^{N}\left(\alpha_{i}-\alpha_{i}^{*}\right)\left(\alpha_{j}-\alpha_{j}^{*}\right)\cdot K\left(X_{i},X_{j}\right) \end{array}$$ (9)

$$\begin{array}{ll} s.t. & \sum_{i=1}^{N}\left(\alpha_{i}-\alpha_{i}^{*}\right)=0;\\ & \alpha_{i},\alpha_{i}^{*}\in \left[0,C\right],\alpha_{i}\cdot \alpha_{i}^{*}=0;\\ & i=1,2,\ldots ,N. \end{array}$$ (10)

where, *K(****X***_*i*_, ***X***_*j*_*) = φ(****X***_*i*_*) φ(****X***_*j*_*)* is called the kernel function. The basic advantage of using a kernel function is avoidance of the problem of seeking and performing mapping *φ(****X****)*. Hence, applying kernel functions gives the solution directly, regardless of the actual mapping. Note that any function that satisfies Mercer’s condition can be used as the kernel function [19]. Finally, the regression function from Eq (6) can formulated explicitly, as:

$$y=f\left(X,\alpha_{i},\alpha_{i}^{*}\right)=\sum_{i=1}^{N}\left(\alpha_{i}-\alpha_{i}^{*}\right)\cdot K\left(X,X_{i}\right)+b$$ (11)

There are various Kernel functions: including linear, radial basis and polynomial. The linear kernel (*K(****X***_*i*_, ***X***_*j*_*) =* ***X***_*i*_ *·* ***X***_*j*_), the simplest, is equivalent to a statistical autoregressive model. The radial basis function, (RBF) kernel (*K(****X***_*i*_, ***X***_*j*_*) = exp(-γ‖****X***_*i*_*—****X***_*j*_*‖*^*2*^*)*, γ > 0 is a free parameter), evaluates the similarity of two samples based on their Euclidian distance, is used to find outliers in a time series, and has proven promising in time series forecasting [27]. The polynomial kernels (*K(****X***_*i*_, ***X***_*j*_*) = (****X***_*i*_ *·* ***X***_*j*_ *+ C)*^*d*^, where integer *d* is the degree of the kernel function, determined before model training) are extremely useful in non-linear data training. Low-degree polynomial kernels tend to save computing time without sacrificing accuracy while high-degree polynomial kernels require more computing time yet cannot promise to increase accuracy. Since *d* = 1 is equivalent to linear kernel, *d* is usually set to 2, and is generally smaller than 5 [28].

### Training and testing datasets

Commercial spring onion seed sales exhibit complex trends that are affected by biological, seasonal and economic factors. However, seed companies usually only have limited datasets for seed demand forecasting. Firstly, only monthly spring onion seed sales data are generally available. Secondly, seed procurement occurs during specific windows of time, as different varieties have different growth cycles (i.e. the planting times in each year; e.g. March, June and September for one variety, and May, August and October for another variety) and seed inventories. Thus, these seasonal trends present as seasonal peaks and valleys in monthly sales data. It is worth noting that different varieties of spring onion may have different seasonal trends. For example, seed variety A may have peak sales between September and November, whereas variety B may have peak sales in March. Thirdly, both seed price and spring onion crop market price influence seed sales. However, the seed prices of different spring onion varieties will remain similar if the seed company has a fixed seed procurement plan [29]. Nevertheless, spring onion seed sales are influenced by fluctuating spring onion crop market prices [30]. Last but not the least, while data on the climate and weather at the production sites, including monthly temperature (average, absolute maximum and minimum) and precipitation are available, the correlations between these meteorological data and seed demand are unclear.

**Fig 2:**
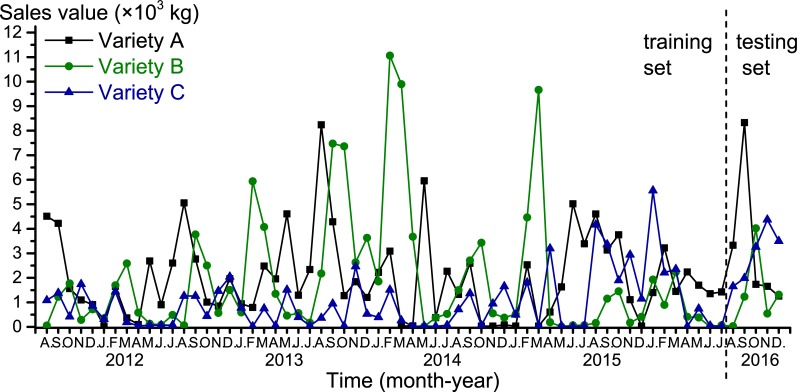
Historical monthly sales data for the three spring onion seed varieties between August 2011 and December 2016.

In this study, monthly sales data for three varieties of spring onion seeds between August 2011 and December 2016 from one of the vegetable seed companies in China are selected for numerical experiments. These three varieties of spring onion combined have covered more than 85% of the sales amount of spring onion seed in the company. The authors have obtained the consent of the company to publicly use these data. The detailed sales data is presented in Fig 2 and S1 Table. The seed inventory data is presented in S2 Table. The varieties are named as A, B and C. The three varieties represent three different types of spring onion seed demand: variety A has high annual demand that increases yearly, variety B has high annual demand that barely increases yearly, and variety C has relatively low annual demand that increases yearly. Since variety A has a different growth cycle (one-year rotation cycle starting in January) to the two other varieties (one-year rotation cycles starting in August), the data from August 2011 to July 2016 were used as a training set and the remaining data (August to December 2016) were employed as the testing set to measure forecasting performance. Standard leave-one-out or 10-fold cross-validation could not be used as the data are time-series based; forecasting of the future could only depend on historical data. The spring onion crop market price data was obtained from the Chinese agriculture information website (jgsb.agri.cn), which monitors the price of agricultural products every month. The meteorological data of Shanghai, where the production sites located, were obtained from the Chinese weather data website (data.cma.cn). These data are listed in S3 Table.

### Hybrid modeling

Although the basic idea of time series forecasting is to investigate patterns in the historical data and predict future trends, variables like sales can be influenced by one or more dynamic factors. To address the influence from dynamic factors, advanced machine learning models are applied. In the case of spring onion seeds, the sales not only follow historical seasonal trends, but may also be influenced by seed inventories and spring onion crop market prices. As statistical-based models only use historical data, their results reflect the linearity and seasonal trends in the data. Inputting the results of statistical-based models into a SVM model is the key point of forecasting using hybrid SVM models.

In the hybrid forecasting model (Fig 1), the monthly sales training set data is inputted into the Holt-Winters model. These statistical-based forecasting results, which reflect the linearity and seasonal trends in the historical data, are then prepared for the SVM model. Then, the results of the Holt-Winters model for the training set and the dynamic factors are inputted into the SVM model as variables. Since there are no general rules for selecting the parameters (*C*, *ε*, *γ*) for the SVM model, the data are learned and the model is tuned to adjust the parameters to optimal values using a grid search method [31]. The starting point and the boundaries of the grid search are determined by a method proposed by Frohlich and Zell [32]. The seasonal trends in the sales data, which would probably be recognized as outliers in statistical-based models, are learned in the SVM model using the RBF kernel. Thus, during training, the RBF kernel is used to obtain the forecasting values. In addition, due to the non-linearity of the sales data, the polynomial kernel (*d* is set to 2) is used to train the SVM model again, and recalculate the results after the initial forecasting values are obtained.

The spring onion seed price, seed inventory and weather data values of the current period are not considered as variables in the hybrid model, as the current values are unknown when forecasting. Therefore, 27 dynamic factor variables, including historical seed sales, seed inventories, spring onion crop market prices and weather data are constructed in the hybrid demand forecasting model (Table 1). For seed inventories and spring onion crop market prices, short-term, mid-term and long-term information is provided by the *variables (t-1) (t-2)*, *MA3 MA6* and *(t-12)*, respectively. Moving averages of the temperature and precipitation data were not calculated, as these data are already average values for the time periods.

**Table 1:** The forecasting performance of the proposed hybrid model with different variables of dynamic factors input.

Variables	MAPE (%) of forecasting without the variable input		
	Variety A	Variety B	Variety C
All variables included ^a^	17.65	49.83	13.35
Seed sales previous month S(t-1) ^b^	43.69	99.72	30.99
Seed sales previous 2 months S(t-2)	45.14	107.38	21.48
Seed sales 3-month moving average S-MA3	57.78	117.07	28.25
Seed sales 6-month moving average S-MA6	43.21	132.33	30.07
Seed sales the same time last year S(t-12)	29.65	83.33	24.05
Seed inventory previous month I(t-1)	21.82	66.78	16.79
Seed inventory previous 2 months I(t-2)	21.37	64.60	16.62
Seed inventory 3-month moving average I-MA3	18.22	44.12	11.46
Seed inventory 6-month moving average I-MA6	16.38	42.12	11.53
Seed inventory the same time last year I(t-12)	18.81	53.95	15.05
Spring onion market price previous month P(t-1)	35.38	91.40	22.72
Spring onion market price previous 2 months P(t-2)	31.28	85.33	22.35
Spring onion market price 3-month moving average P-MA3	41.63	95.11	27.86
Spring onion market price 6-month moving average P-MA6	41.67	110.92	25.14
Spring onion market price the same time last year P(t-12)	29.36	76.73	19.96
Average temperature previous month T(t-1)	23.23	66.41	18.27
Average temperature previous 2 months T(t-2)	23.12	66.09	18.18
Average temperature the same time last year T(t-12)	22.48	64.25	17.68
Absolute max. temperature previous month TX(t-1)	24.64	71.23	20.21
Absolute max. temperature previous 2 months TX(t-2)	24.63	71.19	20.20
Absolute max. temperature the same time last year TX(t-12)	23.39	67.61	19.18
Absolute min. temperature previous month TN(t-1)	24.32	76.65	20.79
Absolute min. temperature previous 2 months TN(t-2)	24.66	77.73	21.09
Absolute min. temperature the same time last year TN(t-12)	26.96	84.98	23.05
Precipitation previous month PC(t-1)	18.09	47.13	12.83
Precipitation previous 2 months PC(t-2)	19.48	54.91	12.36
Precipitation the same time last year PC(t-12)	20.66	53.93	16.79

^a^ This row represents the results of the proposed hybrid model with all variables inputted

^b^ *S(t-1)* refers to seed sales one month before the current month t, S-MA3 = $\sum_{t-1}^{t-3}S(i)/3$, S-MA6 = $\sum_{t-1}^{t-6}S(i)/6$; other variables are expressed in a similar manner.

### Model validation

In order to estimate the performance of the hybrid forecasting model, the results of the ARIMA, mul-HW, RF and ANN forecasting models (as benchmarks) were compared with the proposed hybrid model. For the RF and ANN models, all 27 dynamic factor variables were inputted. To determine the effect of spring onion crop market price, seed inventory and weather data on forecasting accuracy, the results of the proposed hybrid model including different combinations of dynamic factor variables were compared. The Morris method, which analyzes the changes in output due solely to changes in a particular input, was used to estimate the influence and interaction between dynamic factors and the forecasting results [33]. The ARIMA model was run in IBM SPSS Statistics 22.0.0.0 and the other models were run in R version 3.3.1 on an Intel Core i7 PC running at 2.90 GHz with 4 GB memory. For the advanced machine learning models and Morris method, the R packages *tseries*, *e1071*, *neuralnet*, *randomForest* and *sensitivity* were used.

Forecasting performance was evaluated using three error measurements: mean absolute error (MAE), mean squared error (MSE) and mean absolute percentage error (MAPE), defined as:

$$MAE=\frac{1}{n}\sum_{i=1}^{n}\left|F_{i}-A_{i}\right|$$ (12)

$$MSE=\frac{1}{n}\sum_{i=1}^{n}{\left(F_{i}-A_{i}\right)}^{2}$$ (13)

$$MAPE=\frac{1}{n}\sum_{i=1}^{n}\left|\frac{F_{i}-A_{i}}{A_{i}}\right|\times 100\%$$ (14)

where, *F*_*i*_ and *A*_*i*_ represent the forecasting values and actual demand values, respectively. Notice that MAPE is calculated from the ratio of absolute error and the actual value, while MAE and MSE is calculated in terms of the absolute error. This means that MAE and MSE can compare different forecasting methods based on the same data set, whereas MAPE is able to compare the forecasting methods even if different data sets are used.

## Results and discussion

### Forecasting performance of the different models

**Fig 3:**
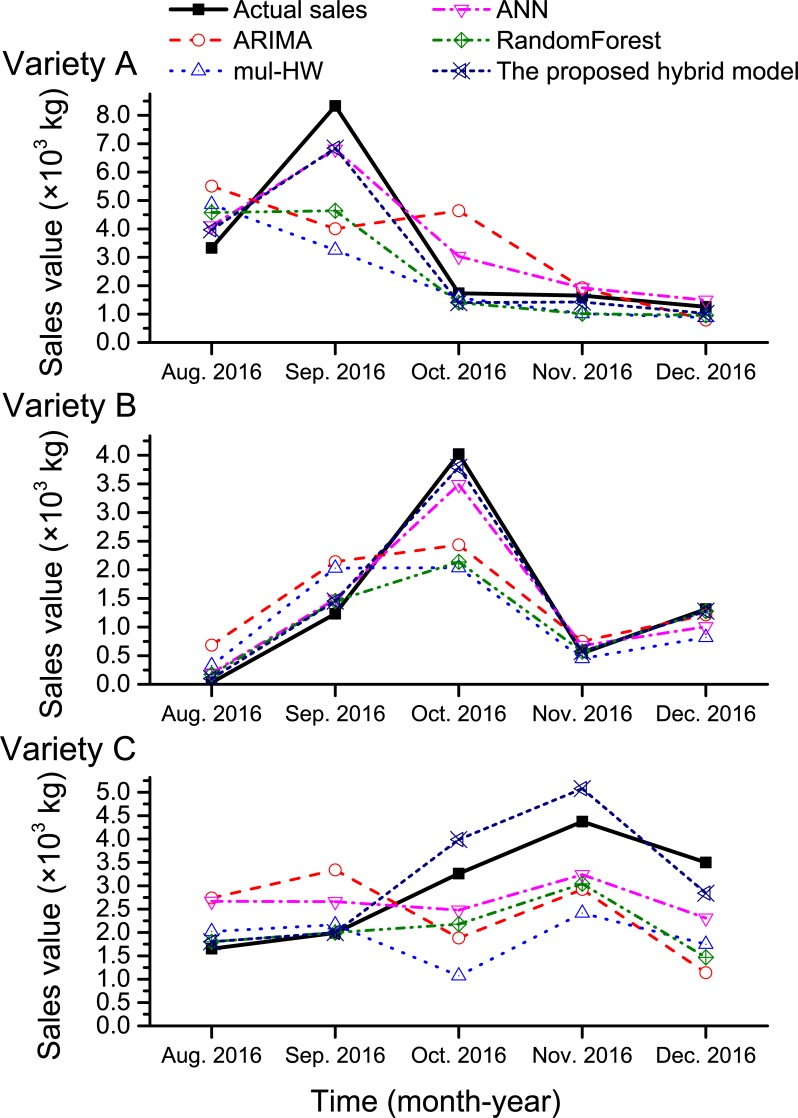
Comparison of actual sales and the forecasting results of the different models in the testing set.

After all of the models were trained on the training set, forecasting was conducted on the testing set and the three error measurements were calculated (Table 2 and Fig 3). As shown in Table 2, the forecasting results for the two time-series models (ARIMA and mul-HW) had larger error measurements than the other three models. The mul-HW model outperformed the ARIMA model for all three seed varieties based on all three error measurements, with the exception that the MAE and MSE values of variety B were lower for the ARIMA model than mul-HW model. The MAPE values of both time-series models were relatively large, in some cases even larger than 100%, suggesting time-series models have unacceptable accuracy for spring onion seed demand forecasting. In addition, the MAPE for variety B was unreasonably high, while the MAPE for variety B excluding the sales value for August 2016 (31.50 kg, mul-HW forecast value was 100.03 kg) was 42.28%, which is close to the MAPE of the two other varieties. This is because the sales value for August 2016 was extremely low compared to the historical sales data (Fig 3), which is a typical example of non-linearity in spring onion seed sales data. Overall, these results suggest that statistical-based models cannot be directly applied for spring onion seed demand forecasting.

**Table 2:** The comparison of demand forecasting performance on the testing set among different models.

	Error type	ARIMA	mul-HW	ANN	RF	Proposed hybrid model
Variety A ^a^	MAPE	67.70%	36.90%	30.27%	32.68%	17.65%
	MAE	289.962	222.280	117.157	177.280	83.2094
	MSE	131271	117031	19305.2	64329.7	11524.3
Variety B	MAPE	446.00%	221.80%	120.92%	104.74%	49.83%
	MAE	98.6893	104.778	39.7028	65.9704	16.7287
	MSE	15583.4	20054.7	1985.59	14737.5	447.259
Variety B (Sep.—Dec. 2016) ^b^	MAPE	39.84%	42.28%	20.32%	18.14%	17.88%
	MAE	114.991	120.396	43.7411	77.3880	18.4626
	MSE	29513.2	24621.7	2343.33	18318.8	535.098
Variety C	MAPE	55.30%	38.60%	35.82%	26.22%	13.35%
	MAE	217.845	184.033	136.930	131.409	64.2900
	MSE	51321.1	48359.8	19578.6	28913.1	6062.61

^a^ The one-year growth cycle of variety A starts in January, while the growth cycles of varieties B and C start in August

^b^ Variety B (Sep.—Dec. 2016) refers to the results for variety B excluding the sales value for August 2016.

Despite the poor performance of the two statistical-based models in processing non-linear data, the results of the mul-HW model reflected the linear trends in the sales data, which provides important information for training of–and forecasting by–the proposed hybrid model. Table 2 compares the forecasting performance of the mul-HW model and proposed hybrid model. The proposed hybrid model had dramatically lower error measurement values than the mul-HW model, indicating the forecasting accuracy of the proposed hybrid model is promising for agricultural production planning and supply chain management in terms of MAPE. Compared to the three advanced machine learning models (ANN, RF, SVM), the proposed hybrid SVM model had the best forecasting performance in terms of all three error measurements. The RF model for demand forecasting consisted of 200 trees with three variables sampled in each tree; the ANN model was constructed with two hidden layers, each having 200 neurons.

This analysis raises three points worth discussing. Firstly, the actual sales value for August 2016 was much lower than the other historical sales values, confirming that extreme variations in spring onion seed sales are possible and do influence the forecasting error of the proposed hybrid model. Secondly, the MAPE values of the RF model, ANN model and proposed hybrid SVM model were higher for variety B than the other two varieties, whereas the MAE values of all three varieties were similar. This suggests that the influence of extreme variation in sales values is non-negligible and needs to be evaluated using other error measurements. However, the MAE and MSE values of the proposed hybrid model were much lower for variety B than the other varieties. Thirdly, with the exception of the sales value for August 2016, the MAPE values of these three models for variety B are close to the MAPE values for the other two varieties. Thus, it is believed that the proposed hybrid model remarkably reduces spring onion seed demand forecasting error, though MAPE may be high–but still acceptable–in some extreme cases.

### SVM parameters on forecasting performance

In order to analyze the influence of SVM parameters on the forecasting performance of the proposed hybrid model, MAPE was selected as the criteria to evaluate the forecasting performance of different parameters, and variety C was selected because it had the lowest MAPE of the three varieties. The candidate parameters are *ε*, *γ* and *C*. However, during training and testing of spring onion seed demand forecasting using the proposed hybrid model, *ε* was found have little effect on forecasting error. Thus, *ε* was set to the default value (*ε* = 0.1).

Table 3 shows the influence of parameters *γ* and *C* on the forecasting performance of the proposed hybrid model for variety C. Generally, when *γ* was larger than 1E-03, MAPE was insensitive to *γ* and *C*. When *γ* was within the range of 1E-06–1E-07 and *C* was larger than 1E+04, MAPE remained within the small range of 13.90 ± 0.60% and was insensitive to the parameters *C* and *γ*. In the numerical experiment, the parameters of the proposed hybrid model were set to *C* = 1E+05, *γ* = 1E-07 and *ε* = 0.1, and the MAPE of the forecasting result was 13.35%. This analysis indicates it is necessary to properly tune these parameters for each specific case, and the grid search method can be used for this process.

**Table 3:** MAPEs of the proposed hybrid model forecasting using different SVM parameters.

*C*	*γ*	MAPE (%) of testing set
1.00E+03	1.00E-05	15.23
	1.00E-06	14.19
	1.00E-07	21.19
	1.00E-08	24.86
1.00E+04	1.00E-05	16.09
	1.00E-06	13.82
	1.00E-07	14.15
	1.00E-08	21.19
***1*.*00E+05***	1.00E-05	17.27
	1.00E-06	14.16
	***1*.*00E-07***	***13*.*35***
	1.00E-08	18.99
1.00E+06	1.00E-05	19.22
	1.00E-06	14.51
	1.00E-07	13.56
	1.00E-08	18.67

### Dynamic factors on forecasting performance

**Fig 4:**
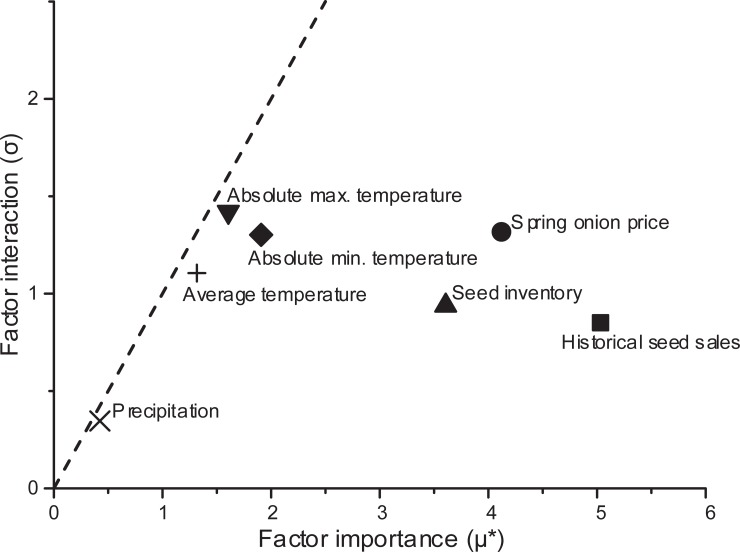
Analysis of the influence and interaction between dynamic factors using the Morris method. The horizontal axis is the mean absolute value of the elementary effect (μ*), which represents the influence of a factor on output. The vertical axis is the standard deviation (*σ*), which represents the interaction of a factor with other factors. The dashed line *σ* = μ* represents point at which interaction with other factors equals the influence on output.

The spring onion crop market price, seed inventory, temperature and precipitation data were inputted into the proposed hybrid model. These dynamic factors provide additional information for demand forecasting beyond the results of the historical sales data. Analysis of the influence and interaction between dynamic factors using the Morris method (*factors* = 6, *r* = 4, where ‘*factors*’ is the number of factors and ‘*r*’ is the number of repetitions) is shown in Fig 4. All dynamic factors were located below the dashed line, indicating the factors influence the output of the model independently–more so than via interaction with other factors. Based on the results of Morris method, it is clear that historical seed sales, spring onion crop market price and seed inventory form a cluster that has the highest importance on seed demand, while the three temperature factors form another cluster with medium importance, and precipitation has the lowest importance.

Since the dynamic factors influence the output independently, the forecasting performance of the proposed hybrid model with individual dynamic factor variables omitted was compared with the results of the original hybrid model that included all 27 variables. In Table 1, each row shows the MAPE values for forecasting using the proposed hybrid model with the individual variables excluded. The first row shows the MAPE value of the model that includes all 27 variables.

With respect to historical seed sales (*S*), omission of *S(t-1)* and *S(t-2)* increased MAPE from 17.64% to 49.89% and 8.13% to 57.55%, respectively. Exclusion of *S-MA3* and *S-MA6* led to the largest increases in MAPE, from 14.90% to 67.24% and 16.72% to 82.50%, respectively. Exclusion of *S(t-12)* increased MAPE from 10.70% to 33.50%. Thus, historical seed sales has a strong influence on forecasting accuracy, with mid-term historical seed sales data having the largest influence.

With respect to seed inventory (*I*), omission of *I(t-1)* and *I(t-2)* led to the largest increases in MAPE, from 3.44% to 16.95% and 3.27% to 14.77%, respectively. Exclusion of *I-MA3* and *I-MA6* did not alter–or even decreased MAPE–and exclusion of *I(t-12)* increased MAPE from 1.16% to 4.12%. Thus, seed inventory has a strong influence on forecasting accuracy, with short-term seed inventory data having the largest influence.

With respect to spring onion crop market price (*P*), exclusion of *P(t-1)* and *P(t-2)* increased MAPE from 9.37% to 41.57% and 9.00% to 35.50%, respectively. Exclusion of *P-MA3* and *P-MA6* led to the largest increases in MAPE, from 14.51% to 45.28% and 11.79% to 61.09%, respectively. Exclusion of *P(t-12)* increased MAPE from 6.61% to 26.90%. In short, spring onion crop market price has a strong influence on forecasting accuracy, with mid-term spring onion crop market price data having the largest influence.

With respect to average temperature (*T*), exclusion of *T(t-1)* increased MAPE from 4.92% to 16.58% Exclusion of *T(t-2)* increased MAPE from 4.83% to 16.26%. Exclusion of *T(t-12)* increased MAPE from 4.33% to 14.42%. Thus, average temperature has limited influence on forecasting accuracy, with short-term average temperature data having the largest influence.

With respect to absolute maximum temperature (*TX*), exclusion of *TX(t-1)* increased MAPE from 6.86% to 21.40%, exclusion of *TX(t-2)* increased MAPE from 6.85% to 21.36%, and exclusion of *TX(t-12)* increased MAPE from 5.74% to 17.78%. Therefore, absolute maximum temperature has limited influence on forecasting accuracy, with the short-term data having the largest influence.

With respect to absolute minimum temperature (*TN*), exclusion of *TN(t-1)* increased MAPE from 6.67% to 26.82%, exclusion of *TN(t-2)* increased MAPE from 7.01% to 27.90%, and exclusion of *TN(t-12)* increased MAPE from 9.31% to 35.15%. In short, absolute minimum temperature has a strong influence on forecasting accuracy; with the long-term data having the largest influence.

With respect to precipitation (*PC*), exclusion of *PC(t-1)* and *PC(t-2)* did not affect–or even decreased–MAPE and exclusion of *PC(t-12)* increased MAPE from 3.01% to 4.10%. In short, precipitation barely has an effect on forecasting accuracy.

Among all these variables, historical seed sales has the largest influence on forecasting error, followed by spring onion crop market price and seed inventory, in agreement with the results of the Morris method. On the other hand, seed inventory, average temperature and absolute maximum temperature have short-term influences on forecasting error and absolute minimum temperature has a long-term influence. Moreover, *I-MA3*, *I-MA6* and the precipitation data could barely influence the forecasting results.

As mentioned in section Training and testing datasets, the three spring onion seed varieties selected for the numerical experiment represent three types of demand, thus the forecasting performance for different seed varieties reflects the performance of the proposed hybrid model for different types of seed demand. As shown in Table 2, the three error measurements of the hybrid model were larger for varieties A and B than variety C. Noting that varieties A and B both have high annual demand, this suggests the proposed hybrid model has better forecasting performance for varieties with low, increasing demand (variety C) than varieties with high, constant demand (varieties A and B). When the data for August 2016 were excluded, the MAPE values for varieties A and B were almost similar and the two other error measurements were similar, indicating the annual demand of a seed variety does not influence the forecasting performance of the proposed hybrid model. Consider a growing seed company with high spring onion seed sales. In order to maintain growth in sales over the long term, it is more important to forecast the trends for varieties that will have increased demand in the future (variety C in this case) than those with high demand at present (varieties A and B). Thus, the proposed hybrid model better forecasts the demand for varieties with low, but increasing, annual demand; thus the hybrid model may help a seed company determine whether a seed variety with low demand will undergo increased demand, and what the increase in demand will be.

## Conclusion

The idea of applying SVM to spring onion seed demand forecasting has been realized in this paper, and a hybrid Holt-Winters and SVM model for spring onion seed demand forecasting is proposed. Our analysis showed the proposed hybrid model outperforms statistical-based models and other two advanced machine learning models, and provides accurate forecasting results for three varieties spring onion seed with different growth cycles and levels of demand. In addition, we discussed the forecasting performance for different varieties when different dynamic factors were used as inputs. Analysis suggested the proposed hybrid model is more promising for forecasting the demand of seed varieties with low, growing annual demand than varieties with high, constant annual demand. Seed inventory, spring onion crop market price, and historical seed sales were the three most important dynamic factors, among which seed inventory has short-term influence while other two have mid-term influence on seed demand forecasting. The absolute minimum temperature is the only dynamic factor having long-term influence. The influence of the parameters of the SVM model on the proposed hybrid model were also explored. The forecasting performance was insensitive to *ε*, while *γ* and *C* play important roles in spring onion seed demand forecasting performance. While the proposed forecasting model is based on spring onion seed production, it could also be applied to other crop seeds whose demand is also influenced by dynamic factors such as historical sales, inventories, crop market prices and weather conditions, etc.

In real life, it is difficult to predict and consider the growers’ reaction to a specific event such as an increase in crop market prices or extreme weather events, either of which may be the reason for the sudden reduction in the sale of variety B in August, 2016. To explore this point, future works should focus on additional factors, such as weather and region. However, the key findings of this paper support the use of a hybrid SVM model for high accuracy demand forecasting to guide decision-making processes in sustainable agricultural production.

## Supporting information

S1 Table Historical monthly sales data (kg) of the three spring onion seed varieties. (DOCX)

S2 Table Historical seed inventory data (kg) of the three spring onion seed varieties. (DOCX)

S3 Table Spring onion market price, temperature and precipitation data. (DOCX)
