# Supplementary material for: Spring onion seed demand forecasting using a hybrid Holt-Winters and support vector machine model
Source: PLoS One. 2019 Jul 25;14(7):e0219889. doi: 10.1371/journal.pone.0219889 (PMC6658075; doi:10.1371/journal.pone.0219889)
Supplement: S1 Table — (DOCX) [file pone.0219889.s001.docx]

S1 Table. Historical monthly sales data (kg) of the three spring onion seed varieties.

| Year | Month | Variety A | Variety B | Variety C |  | Year | Month | Variety A | Variety B | Variety C |
| --- | --- | --- | --- | --- | --- | --- | --- | --- | --- | --- |
| 2011 | Aug. | 4509.40 | 56.42 | 1084.72 |  | 2014 | Apr. | 41.30 | 3677.31 | 22.89 |
| 2011 | Sep. | 4222.75 | 1227.59 | 1385.09 |  | 2014 | May. | 5956.02 | 19.53 | 33.95 |
| 2011 | Oct. | 1561.42 | 1772.12 | 418.88 |  | 2014 | Jun. | 378.00 | 378.00 | 22.75 |
| 2011 | Nov. | 1097.18 | 286.72 | 1743.70 |  | 2014 | Jul. | 2267.37 | 529.83 | 69.30 |
| 2011 | Dec. | 907.41 | 718.41 | 853.44 |  | 2014 | Aug. | 1323.00 | 1498.14 | 713.79 |
| 2012 | Jan. | 35.00 | 346.50 | 315.00 |  | 2014 | Sep. | 2597.49 | 2709.63 | 1366.89 |
| 2012 | Feb. | 1654.38 | 1695.75 | 1435.14 |  | 2014 | Oct. | 35.00 | 3431.26 | 63.00 |
| 2012 | Mar. | 367.29 | 2586.36 | 189.00 |  | 2014 | Nov. | 35.00 | 551.88 | 946.89 |
| 2012 | Apr. | 98.35 | 585.27 | 40.95 |  | 2014 | Dec. | 79.10 | 378.00 | 1652.49 |
| 2012 | May. | 2687.58 | 129.78 | 69.79 |  | 2015 | Jan. | 35.00 | 521.01 | 495.81 |
| 2012 | Jun. | 903.42 | 84.00 | 66.36 |  | 2015 | Feb. | 2530.08 | 4462.71 | 1806.84 |
| 2012 | Jul. | 2603.16 | 487.13 | 74.69 |  | 2015 | Mar. | 35.00 | 9663.22 | 47.81 |
| 2012 | Aug. | 5056.38 | 62.37 | 1260.00 |  | 2015 | Apr. | 605.64 | 189.00 | 3197.88 |
| 2012 | Sep. | 2768.85 | 3769.64 | 1260.00 |  | 2015 | May. | 1630.44 | 7.20 | 40.32 |
| 2012 | Oct. | 1008.00 | 2502.36 | 441.00 |  | 2015 | Jun. | 5018.58 | 64.26 | 16.94 |
| 2012 | Nov. | 848.05 | 570.15 | 1464.75 |  | 2015 | Jul. | 3388.14 | 68.11 | 38.29 |
| 2012 | Dec. | 1964.34 | 1505.70 | 2044.35 |  | 2015 | Aug. | 4604.04 | 160.02 | 4165.56 |
| 2013 | Jan. | 939.96 | 592.06 | 776.79 |  | 2015 | Sep. | 3134.25 | 1152.20 | 3360.42 |
| 2013 | Feb. | 795.06 | 5935.30 | 18.90 |  | 2015 | Oct. | 3756.06 | 1452.15 | 1894.41 |
| 2013 | Mar. | 2480.31 | 4073.02 | 751.59 |  | 2015 | Nov. | 1108.80 | 174.79 | 2940.21 |
| 2013 | Apr. | 1957.41 | 1348.90 | 48.30 |  | 2015 | Dec. | 35.00 | 413.56 | 1146.60 |
| 2013 | May. | 4605.93 | 461.02 | 1512.00 |  | 2016 | Jan. | 1399.37 | 1930.46 | 5558.77 |
| 2013 | Jun. | 1291.50 | 562.94 | 394.38 |  | 2016 | Feb. | 3220.00 | 899.29 | 2212.00 |
| 2013 | Jul. | 2339.19 | 173.25 | 50.89 |  | 2016 | Mar. | 1446.90 | 2233.07 | 2355.99 |
| 2013 | Aug. | 8238.51 | 2176.02 | 365.40 |  | 2016 | Apr. | 2239.30 | 413.28 | 13.44 |
| 2013 | Sep. | 4284.00 | 7471.80 | 948.15 |  | 2016 | May. | 1701.00 | 382.41 | 744.10 |
| 2013 | Oct. | 1273.30 | 7362.18 | 36.54 |  | 2016 | Jun. | 1352.40 | 10.10 | 52.22 |
| 2013 | Nov. | 1840.93 | 2630.39 | 2465.19 |  | 2016 | Jul. | 1426.25 | 66.78 | 25.55 |
| 2013 | Dec. | 1201.55 | 3629.78 | 529.83 |  | 2016 | Aug. | 3329.20 | 31.50 | 1652.00 |
| 2014 | Jan. | 2226.42 | 1852.90 | 392.00 |  | 2016 | Sep. | 8329.19 | 1232.18 | 1989.40 |
| 2014 | Feb. | 3089.52 | 11059.93 | 1512.00 |  | 2016 | Oct. | 1734.60 | 4019.89 | 3260.56 |
| 2014 | Mar. | 35.00 | 9895.83 | 266.00 |  | 2016 | Nov. | 1653.05 | 545.44 | 4372.20 |
|  |  |  |  |  |  | 2016 | Dec. | 1257.20 | 1313.55 | 3498.60 |
