# Supplementary material for: Spring onion seed demand forecasting using a hybrid Holt-Winters and support vector machine model
Source: PLoS One. 2019 Jul 25;14(7):e0219889. doi: 10.1371/journal.pone.0219889 (PMC6658075; doi:10.1371/journal.pone.0219889)
Supplement: S2 Table — (DOCX) [file pone.0219889.s002.docx]

S2 Table. Historical seed inventory data (kg) of the three spring onion seed varieties.

| Year | Month | Variety A | Variety B | Variety C |  | Year | Month | Variety A | Variety B | Variety C |
| --- | --- | --- | --- | --- | --- | --- | --- | --- | --- | --- |
| 2011 | Aug. | 19990.60 | 27943.58 | 11795.28 |  | 2014 | Apr. | 8067.15 | 11111.10 | 6919.29 |
| 2011 | Sep. | 15767.85 | 26715.99 | 10410.19 |  | 2014 | May. | 9111.13 | 11091.57 | 6885.34 |
| 2011 | Oct. | 14206.43 | 24943.87 | 9991.31 |  | 2014 | Jun. | 8733.13 | 10713.57 | 6862.59 |
| 2011 | Nov. | 13109.25 | 24657.15 | 12062.61 |  | 2014 | Jul. | 9965.76 | 10183.74 | 6793.29 |
| 2011 | Dec. | 12201.84 | 23938.74 | 11209.17 |  | 2014 | Aug. | 8642.76 | 8685.60 | 6079.50 |
| 2012 | Jan. | 12166.84 | 35492.24 | 10894.17 |  | 2014 | Sep. | 6045.27 | 5975.97 | 6462.61 |
| 2012 | Feb. | 10512.46 | 33796.49 | 9459.03 |  | 2014 | Oct. | 34010.27 | 2544.71 | 6399.61 |
| 2012 | Mar. | 10145.17 | 34710.13 | 9270.03 |  | 2014 | Nov. | 33975.27 | 1992.83 | 5452.72 |
| 2012 | Apr. | 10046.82 | 34124.86 | 9229.08 |  | 2014 | Dec. | 40896.17 | 22614.83 | 5900.23 |
| 2012 | May. | 7359.24 | 33995.08 | 13149.29 |  | 2015 | Jan. | 40861.17 | 22093.82 | 5404.42 |
| 2012 | Jun. | 6455.82 | 33911.08 | 13082.93 |  | 2015 | Feb. | 38331.09 | 17631.11 | 3597.58 |
| 2012 | Jul. | 3852.66 | 33423.95 | 13008.24 |  | 2015 | Mar. | 38296.09 | 7967.89 | 3549.77 |
| 2012 | Aug. | 5796.28 | 33361.58 | 11748.24 |  | 2015 | Apr. | 37690.45 | 7778.89 | 351.89 |
| 2012 | Sep. | 3027.43 | 29591.94 | 10488.24 |  | 2015 | May. | 43060.01 | 7771.89 | 14311.57 |
| 2012 | Oct. | 23019.43 | 27089.58 | 10047.24 |  | 2015 | Jun. | 38041.43 | 7707.63 | 14294.63 |
| 2012 | Nov. | 22171.38 | 26519.43 | 13132.49 |  | 2015 | Jul. | 34653.29 | 7639.52 | 14256.34 |
| 2012 | Dec. | 20207.04 | 25013.73 | 11088.14 |  | 2015 | Aug. | 30049.25 | 7479.50 | 10090.78 |
| 2013 | Jan. | 19267.08 | 24421.67 | 10311.35 |  | 2015 | Sep. | 26915.00 | 6327.30 | 6730.36 |
| 2013 | Feb. | 18472.02 | 18486.37 | 10292.45 |  | 2015 | Oct. | 26658.94 | 8375.15 | 4835.95 |
| 2013 | Mar. | 15991.71 | 14413.35 | 9540.86 |  | 2015 | Nov. | 25550.14 | 8200.36 | 1895.74 |
| 2013 | Apr. | 14034.30 | 13064.45 | 9492.56 |  | 2015 | Dec. | 29015.14 | 14786.80 | 14749.14 |
| 2013 | May. | 16428.37 | 16103.43 | 10962.56 |  | 2016 | Jan. | 27615.77 | 12856.34 | 9190.37 |
| 2013 | Jun. | 15136.87 | 15540.49 | 10568.18 |  | 2016 | Feb. | 24395.77 | 11957.05 | 6978.37 |
| 2013 | Jul. | 19797.68 | 22367.24 | 10517.29 |  | 2016 | Mar. | 22948.87 | 9723.98 | 4622.38 |
| 2013 | Aug. | 11559.17 | 20191.22 | 10151.89 |  | 2016 | Apr. | 20709.57 | 9310.70 | 4608.94 |
| 2013 | Sep. | 7275.17 | 12719.42 | 9203.74 |  | 2016 | May. | 26008.57 | 8928.29 | 12264.84 |
| 2013 | Oct. | 13001.87 | 12357.24 | 9167.20 |  | 2016 | Jun. | 24656.17 | 8921.29 | 12212.62 |
| 2013 | Nov. | 11160.94 | 9726.85 | 9642.01 |  | 2016 | Jul. | 26729.92 | 15854.51 | 12187.07 |
| 2013 | Dec. | 13459.39 | 27097.07 | 9112.18 |  | 2016 | Aug. | 23400.72 | 15823.01 | 10535.07 |
| 2014 | Jan. | 11232.97 | 25244.17 | 8720.18 |  | 2016 | Sep. | 15071.53 | 14590.83 | 15545.67 |
| 2014 | Feb. | 8143.45 | 14184.24 | 7208.18 |  | 2016 | Oct. | 16836.93 | 17570.94 | 12285.11 |
| 2014 | Mar. | 8108.45 | 14788.41 | 6942.18 |  | 2016 | Nov. | 15183.88 | 17025.50 | 7912.91 |
|  |  |  |  |  |  | 2016 | Dec. | 13926.68 | 15711.95 | 4414.31 |
