# Supplementary material for: Spring onion seed demand forecasting using a hybrid Holt-Winters and support vector machine model
Source: PLoS One. 2019 Jul 25;14(7):e0219889. doi: 10.1371/journal.pone.0219889 (PMC6658075; doi:10.1371/journal.pone.0219889)
Supplement: S3 Table — (DOCX) [file pone.0219889.s003.docx]

S3 Table. Spring onion market price, temperature and precipitation data.

| Year | Month | Spring onion market price(￥/kg) ^a^ | T. ave.(℃) | T. abs. max(℃) | T. abs. min(℃) | Prec.(mm) |
| --- | --- | --- | --- | --- | --- | --- |
| 2011 | Aug. | 2.13 | 28.2 | 35.5 | 22.7 | 308.8 |
| 2011 | Sep. | 2.48 | 24.6 | 33.9 | 14.4 | 27.8 |
| 2011 | Oct. | 2.10 | 19.2 | 26.5 | 11.7 | 48.3 |
| 2011 | Nov. | 1.65 | 16.6 | 26.0 | 4.3 | 41.5 |
| 2011 | Dec. | 1.73 | 6.6 | 15.3 | -2.5 | 27.6 |
| 2012 | Jan. | 3.21 | 4.8 | 10.8 | -5.0 | 49.3 |
| 2012 | Feb. | 3.03 | 4.5 | 11.9 | -3.7 | 100.3 |
| 2012 | Mar. | 4.54 | 9.7 | 25.3 | 0.6 | 116.7 |
| 2012 | Apr. | 3.54 | 17.8 | 28.5 | 7.7 | 59.2 |
| 2012 | May. | 3.15 | 21.4 | 34.1 | 14.9 | 132.5 |
| 2012 | Jun. | 2.99 | 24.9 | 36.1 | 19.5 | 80.8 |
| 2012 | Jul. | 2.83 | 30.1 | 38.3 | 22.7 | 109.5 |
| 2012 | Aug. | 2.46 | 29.5 | 36.0 | 23.0 | 168.1 |
| 2012 | Sep. | 2.49 | 24.0 | 31.9 | 15.5 | 80.3 |
| 2012 | Oct. | 2.02 | 19.8 | 27.4 | 10.1 | 23.1 |
| 2012 | Nov. | 1.88 | 12.1 | 20.1 | 1.5 | 120.4 |
| 2012 | Dec. | 2.55 | 6.2 | 17.8 | -4.4 | 87.4 |
| 2013 | Jan. | 3.24 | 5.0 | 18.0 | -3.1 | 46.7 |
| 2013 | Feb. | 3.71 | 6.8 | 17.8 | -2.3 | 66.6 |
| 2013 | Mar. | 2.34 | 11.5 | 30.9 | 0.1 | 52.6 |
| 2013 | Apr. | 2.50 | 15.6 | 32.3 | 3.2 | 74.6 |
| 2013 | May. | 2.78 | 21.5 | 31.3 | 9.4 | 125.2 |
| 2013 | Jun. | 2.32 | 24.2 | 36.1 | 15.8 | 181.5 |
| 2013 | Jul. | 2.19 | 32.2 | 39.0 | 23.7 | 102.8 |
| 2013 | Aug. | 2.36 | 31.7 | 39.9 | 24.2 | 73.1 |
| 2013 | Sep. | 2.76 | 25.2 | 35.0 | 16.0 | 56.3 |
| 2013 | Oct. | 2.78 | 20.0 | 28.7 | 8.9 | 291.7 |
| 2013 | Nov. | 2.73 | 13.5 | 27.8 | -1.7 | 20.4 |
| 2013 | Dec. | 2.93 | 6.3 | 19.7 | -4.2 | 50.5 |
| 2014 | Jan. | 2.94 | 7.0 | 20.2 | -4.1 | 21.1 |
| 2014 | Feb. | 3.18 | 6.3 | 24.5 | -2.4 | 155.9 |
| 2014 | Mar. | 2.86 | 11.7 | 24.5 | 1.6 | 46.7 |
| 2014 | Apr. | 2.52 | 16.0 | 24.8 | 4.6 | 139.4 |
| 2014 | May. | 2.20 | 22.0 | 34.0 | 9.2 | 61.8 |
| 2014 | Jun. | 1.96 | 23.6 | 32.7 | 18.6 | 175.8 |
| 2014 | Jul. | 2.00 | 27.7 | 35.0 | 20.6 | 209.6 |
| 2014 | Aug. | 2.27 | 26.6 | 35.2 | 19.6 | 271.4 |
| 2014 | Sep. | 2.22 | 24.5 | 31.8 | 18.2 | 136.6 |
| 2014 | Oct. | 2.22 | 20.3 | 30.4 | 8.9 | 37.3 |
| 2014 | Nov. | 1.84 | 14.7 | 23.0 | 4.4 | 35.2 |
| 2014 | Dec. | 1.98 | 5.8 | 15.6 | -3.5 | 5.2 |

Continued on next page.

S3 Table. Continues.

| Year | Month | Spring onion market price(￥/kg) ^a^ | T. ave.(℃) | T. abs. max(℃) | T. abs. min(℃) | Prec.(mm) |
| --- | --- | --- | --- | --- | --- | --- |
| 2015 | Jan. | 1.72 | 6.3 | 19.7 | -4.8 | 61.1 |
| 2015 | Feb. | 1.89 | 7.0 | 18.4 | -3.8 | 81.2 |
| 2015 | Mar. | 1.80 | 10.9 | 29.0 | -1.2 | 96.4 |
| 2015 | Apr. | 1.67 | 16.0 | 31.7 | 3.7 | 108.9 |
| 2015 | May. | 1.74 | 20.7 | 29.5 | 10.6 | 131.4 |
| 2015 | Jun. | 2.80 | 24.6 | 35.0 | 17.2 | 519.0 |
| 2015 | Jul. | 3.29 | 27.0 | 38.1 | 16.9 | 140.5 |
| 2015 | Aug. | 3.16 | 28.2 | 38.5 | 21.9 | 125.6 |
| 2015 | Sep. | 3.14 | 24.4 | 30.9 | 18.3 | 146.2 |
| 2015 | Oct. | 2.69 | 19.6 | 27.1 | 10.2 | 48.1 |
| 2015 | Nov. | 2.69 | 14.0 | 26.2 | -2.1 | 109.3 |
| 2015 | Dec. | 3.40 | 7.9 | 17.9 | -1.7 | 81.5 |
| 2016 | Jan. | 4.21 | 4.7 | 17.3 | -7.8 | 80.8 |
| 2016 | Feb. | 6.90 | 7.1 | 20.9 | -3.5 | 21.1 |
| 2016 | Mar. | 6.85 | 11.2 | 25.3 | -1.8 | 49.3 |
| 2016 | Apr. | 6.18 | 17.0 | 27.8 | 8.3 | 142.7 |
| 2016 | May. | 5.22 | 20.8 | 29.9 | 11.6 | 196.1 |
| 2016 | Jun. | 3.49 | 24.4 | 34.7 | 17.3 | 203.1 |
| 2016 | Jul. | 2.68 | 30.3 | 39.2 | 23.0 | 166.4 |
| 2016 | Aug. | 2.36 | 29.7 | 36.0 | 19.7 | 32.3 |
| 2016 | Sep. | 2.53 | 24.8 | 33.9 | 12.0 | 291.7 |
| 2016 | Oct. | 2.35 | 21.0 | 31.9 | 13.9 | 295.9 |
| 2016 | Nov. | 2.48 | 13.7 | 24.1 | 1.2 | 68.9 |
| 2016 | Dec. | 2.54 | 9.2 | 19.7 | -1.8 | 48.9 |

a ￥:1 RMB￥(Renminbi Yuan) = US$ 0.1446.
